# 

**Published:** 1890-08-16

**Authors:** 


SATURDAY, AUGUST 16th, 1890.

**Abroad and at Home** - 289

**Words of Consolation.**—Unbelief - 290

**The Doctor's Pulpit.**—Race Rivalries - 291

**The Moral Basis of Private Property.** II.—The Good of the Community - 292

**Working Women in Large Towns.** VII.—Matchbox, Brush, and Box Makers - 293

**General Charities** - 294

**Turning Over New Leaves.**—"Hospitals" in Chambers' Encyclopædia - 294

**Editor's Letter-Box** - 294

**Everybody's Page** - 295

**Notes and News** - 296

**Annotations.**—Execution by Electricity—"A Reprehensible Practice"—The Wrongs of Private Nurses - 298

**The Practitioner's Mirror and Retrospect:**—

MEDICINE—A New Name, if Not a New Disease, 299; Hemiplegia in Pneumonia of Children - 300

SURGERY—Insect Stings, 300; Goitre Treated by Electricity—Treatment of Fractured Legs - 301

NEW DRUGS, APPLIANCES, AND THINGS MEDICAL—Florador, 301; Alfred Carter's Invalid Furniture, 302; The "Doris" Safety Belt for Children - 303

**Students' Column** - 303

**Passing Topics**—Frivolity's Labours - 304

**Scraps and Gleanings** - 304

EXTRA SUPPLEMENT—THE NURSING MIRROR.

**En Passant**—Neatly Put—Short Items—Asylum Attendants—Pay or be Paid?—A Word of Caution—A Strike at Melbourne - lxxxiii

**Dr. Tarrant's Mistake.**—(*Concluded*) - lxxxiv

**Life and High Life at Johannesburg** - lxxxv

**Everybody's Opinion.**—Miss Gee's Home, Montreal, lxxxv; Nursing at the London—A Home of Rest - lxxxvi

**Notes and Queries** - lxxxvi

**Vacancies** - lxxxvi

**Amusements and Relaxation** - lxxxvi

